# An Updated Review of Combined Hepatocellular Cholangiocarcinoma: A Rare and Poorly Understood Neoplasm

**DOI:** 10.3390/diagnostics16020314

**Published:** 2026-01-19

**Authors:** Gavin Low, Xu Jing Qian, Ali Ramji, Blaire Anderson, Safwat Girgis, Karim Samji, Mitchell P. Wilson

**Affiliations:** 1 Department of Radiology & Diagnostic Imaging, University of Alberta Hospital, University of Alberta, WMC 2B2.41 8440-112 ST, Edmonton, AB T6G2B7, Canada; xqian@ualberta.ca (X.J.Q.); aramji@ualberta.ca (A.R.); ksamji@ualberta.ca (K.S.); mitch.wilson@ualberta.ca (M.P.W.); 2 Department of Surgery, University of Alberta, Edmonton, AB T6G2B7, Canada; blanders@ualberta.ca; 3 Department of Laboratory Medicine & Pathology, University of Alberta, Edmonton, AB T6G2B7, Canada; sgirgis@ualberta.ca

**Keywords:** combined hepatocellular cholangiocarcinoma, magnetic resonance imaging, computed tomography, treatment options, prognosis, radiomics

## Abstract

Combined hepatocellular cholangiocarcinoma (cHCC-CC) is a rare and poorly understood primary liver cancer. First identified over a century ago, it has been referred to by various names and reclassified multiple times since the initial description. Diagnosis is extremely challenging as the tumor can mimic hepatocellular carcinoma (HCC) or intrahepatic cholangiocarcinoma (ICC) on imaging or show overlapping features of both. The tumor may also be incorrectly diagnosed with biopsy due to inadequate tissue sampling. As such, many tumors are only correctly diagnosed histologically following surgical resection or transplantation for presumptive HCC. A variety of treatment options are available, although no national or international consensus exists regarding the optimal treatment strategy. Treatment outcomes vary with cHCC-CC showing an intermediate prognosis between HCC and ICC. In this updated review, we provide a conceptual overview of this intriguing neoplasm, including its classification and origins, epidemiology, clinical characteristics, and diagnostic and treatment options. Finally, we discuss the use of radiomics artificial intelligence (AI) to address challenges in lesion differentiation from HCC and ICC, and in predicting post-treatment survival and recurrence.

## 1. Introduction

Combined hepatocellular cholangiocarcinoma (cHCC-CC) is a rare and poorly understood primary liver cancer. First discovered over a century ago, it has been referred to by various names and has undergone multiple reclassifications. The tumor has a biphenotypic composition featuring varying proportions of hepatocellular carcinoma (HCC) and intrahepatic cholangiocarcinoma (ICC). This unique dual tumor characteristic leads to variability in imaging findings that may mimic HCC or ICC, as well as differences in biologic behavior and tumor aggressiveness. In this updated review, we provide a conceptual overview of this intriguing neoplasm, covering its classification and origins, epidemiology, clinical characteristics, and diagnostic and treatment options. Finally, we discuss the role of radiomics artificial intelligence (AI) in addressing challenges in lesion differentiation from HCC and ICC, and in predicting post-treatment survival and recurrence.

## 2. Nomenclature, Classifications and Origins

**Figure 1:**
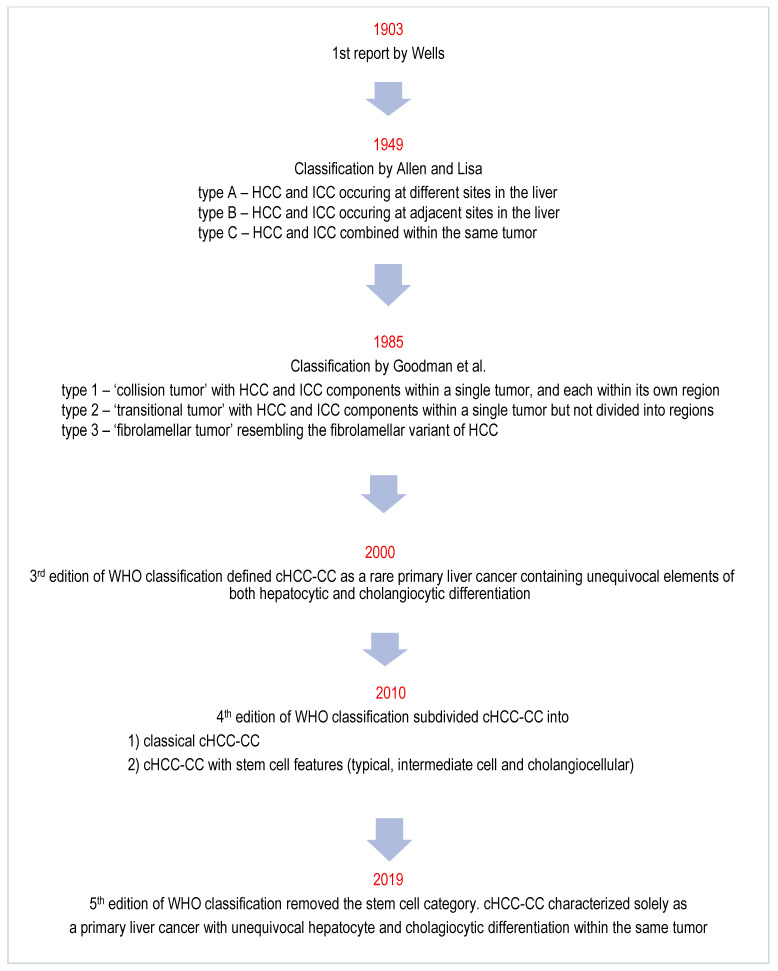
Historical Milestones in cHCC-CC Classification [3,4,5].

A variety of alternative terminologies have been used for cHCC-CC in the medical literature, including biphenotypic liver tumor/cancer, mixed hepatocellular cholangiocarcinoma, mixed HCC-CC, cholangiohepatoma and combined liver and bile duct carcinoma [1,2]. Definitions and classifications have evolved over time, with notable contributions by Wells [3], Allen and Lisa [4], Goodman et al. [5], along with the World Health Organization (WHO) classifications from 2000 [6], 2010 [7] and 2019 [8] [Figure 1]. The origins of cHCC-CC are unclear, with several theories proposed [2]. First, that HCC and ICC develop independently of each other [2]. Second, that cHCC-CC originates from stem/progenitor cells that differentiate into both hepatocytic and cholangiocytic components [2,9]. Third, that HCC arises first and then variably transforms into ICC [2,4].

## 3. Epidemiology and Clinical Characteristics

cHCC-CC accounts for 0.4 to 14.2% of all primary liver malignancies [10,11,12]. According to data from the Surveillance, Epidemiology, and End Results registry, cHCC-CC has an overall incidence of 0.05 per 100,000 per year [13]. Males are involved in 69.4%, with a male vs. female incidence of 0.08 vs. 0.03 per 100,000 per year [13]. Median age at diagnosis is 62.5 ± 12 years [13]. Incidence increases with age, peaking at 60 to 64 years in men and 75 to 79 years in women [13]. Among racial groups, cHCC-CC is most common in Asians and Pacific islanders, followed equally by Caucasians and Blacks, and is lowest in the Indigenous population. While the specific etiology for cHCC-CC is unknown, it shares common risk factors with HCC and ICC [12,14,15]. This includes hepatitis B and C, cirrhosis, alcohol consumption and metabolic syndrome [14,16]. Additionally, cHCC-CC may occur de novo without pre-existing liver disease [17,18]. Clinical features are non-specific and can lead to a late presentation [2,14,19]. Symptoms include abdominal discomfort, weight loss, fatigue, fever, and itching [18]. Signs in advanced disease include jaundice, hepatomegaly, ascites, and cholangitis [14]. A raised serum alpha fetoprotein (AFP) > 20 ng/mL, typically associated with HCC, is seen in 58.3% of cHCC-CCs compared to 66.5% of HCCs and 13.7% of ICCs [18,20]. An elevated serum carbohydrate antigen 19-9 (CA19-9), typically associated with ICC, is observed in 28.9% of cHCC-CCs compared to 40% of ICCs and 2.2% of HCCs [18,21]. Simultaneous elevations of serum AFP and CA19-9 occur in 15.6 to 17.8% of cHCC-CCs [18,21,22]. At clinical presentation, cHCC-CC are poorly differentiated in 30.8%, moderately differentiated in 19.1%, well differentiated in 3.2%, and undifferentiated in 3% [13]. The most common stage at presentation is stage 4 (26.8%) [13]. cHCC-CC has a biologic behavior and prognosis that is intermediate between HCC and ICC [2,16,20,23]. Vascular invasion (a characteristic of HCC) and hilar nodal metastases (a characteristic of ICC) occur in 38.5% and 12 to 33% of cHCC-CCs, respectively [24]. cHCC-CC has a survival rate of 43.4% at 1 year, 21.5% at 3 years, and 17.1% at 5 years [23].

## 4. Diagnosis

**Figure 2:**
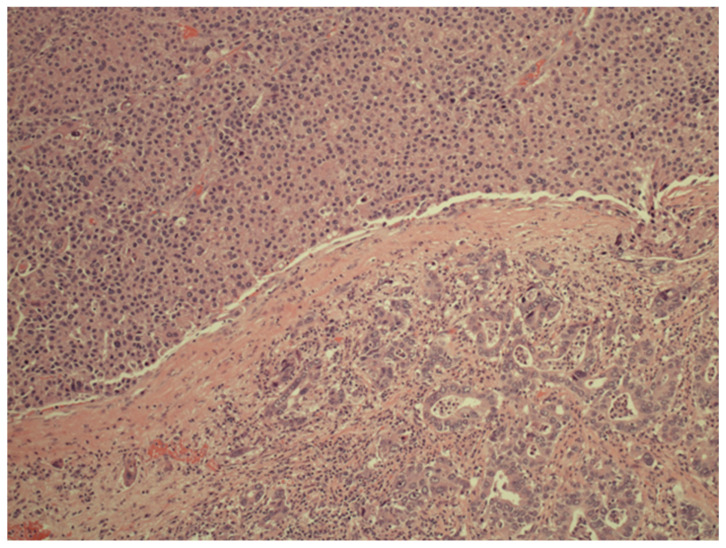
A histology image of a liver resection specimen (hematoxylin and eosin staining at 100× total magnification). The top half of the image shows abnormal hepatocytic proliferation with expanded trabeculae consistent with a HCC component. The lower half shows atypical glandular crowded proliferation consistent with an ICC component. Overall findings are diagnostic of cHCC-CC.

The diagnostic gold standard is histopathology. Obtaining representative tissue is essential. If samples capture only one of the cHCC-CC components, the tumor may be misdiagnosed as HCC, ICC, or even metastatic adenocarcinoma [25]. Resection specimens must be meticulously reviewed [Figure 2]. Percutaneous needle biopsy has the downside of only being able to sample a small portion of the tumor, with a reported sensitivity of 11.1% to 48% for cHCC-CC [14,25,26]. Performing multiple core biopsies from different regions of the tumor is recommended to improve diagnostic accuracy and avoid incorrect diagnosis [18]. The accurate assessment of the actual prevalence of cHCC-CC can be significantly influenced by the availability and timeliness of adequate histopathological evaluations. In patients with underlying cirrhosis, epidemiological data from recent years have been notably impacted by restrictions related to the COVID-19 pandemic. According to the recent CERO-19 international study, which involved 76 centers, approximately 41% of these centers modified their diagnostic procedures, while 80.9% altered their screening programs. These changes in clinical practice due to the pandemic have led to delayed diagnoses, reduced surveillance programs, and a decrease in invasive diagnostic procedures, such as liver biopsies, ultimately impairing patient outcomes [27].

As per LI-RADS 2018, HCC and ICC correspond to LR 4/5 and LR M imaging features on computed tomography (CT) and magnetic resonance imaging (MRI), respectively [28]. These are detailed in Table 1. On CT/MRI, HCC typically shows non-rim arterial phase hyperenhancement followed by portal/delayed phase washout [28]. In comparison, ICC shows rim arterial phase hyperenhancement followed by progressive central enhancement [28]. LR 4/5 observations can be managed as HCC without biopsy, whereas LR M observations require biopsy confirmation [28].

**Figure 3:**
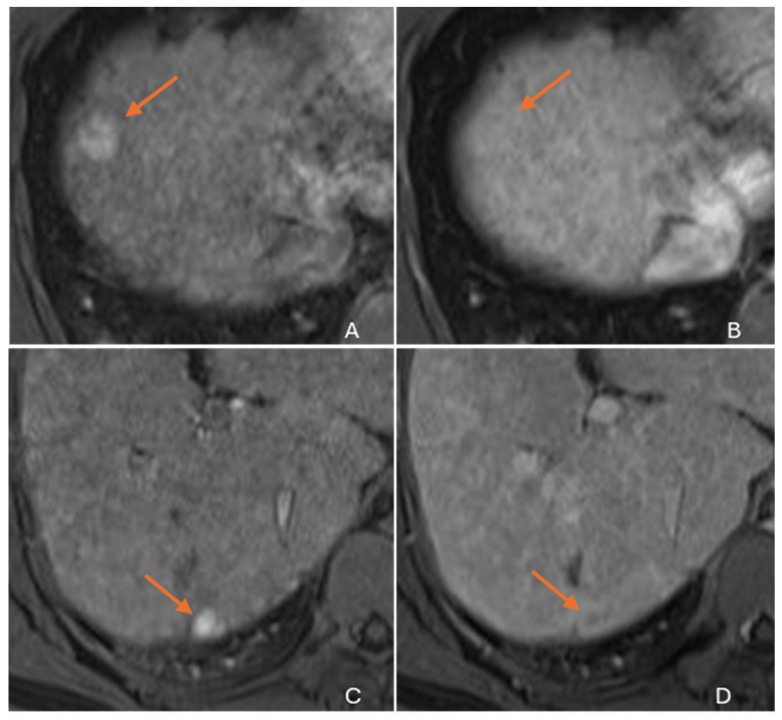
Axial gadolinium-enhanced MRI images in a 43-year-old male with HCV cirrhosis and pathologically proven cHCC-CCs on explant. A 2 cm cHCC-CC (**arrows**) at the anterior subcapsular border of segment 8 shows LR 4 features with diffuse arterial hyperenhancement (**A**) and is isosignal on the delayed phase (**B**). At a lower slice, a 1.2 cm cHCC-CC (**arrows**) at the posterior subcapsular border of segment 7/6 shows LR 5 features with diffuse arterial hyperenhancement (**C**) followed by delayed phase washout (**D**).

**Figure 4:**
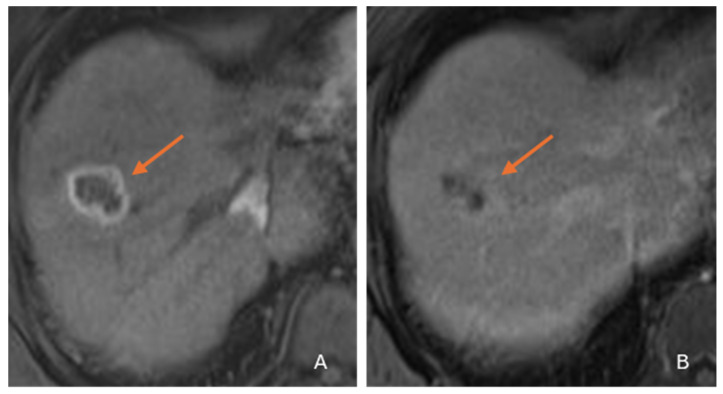
Axial gadolinium-enhanced MRI images in a 55-year-old male with cirrhosis from alcohol use disorder, and a pathologically proven cHCC-CC on biopsy. A 3.1 cm cHCC-CC (**arrows**) in segment 8 shows LR M features with peripheral rim arterial hyperenhancement (**A**) followed by central progression on the delayed phase (**B**).

**Figure 5:**
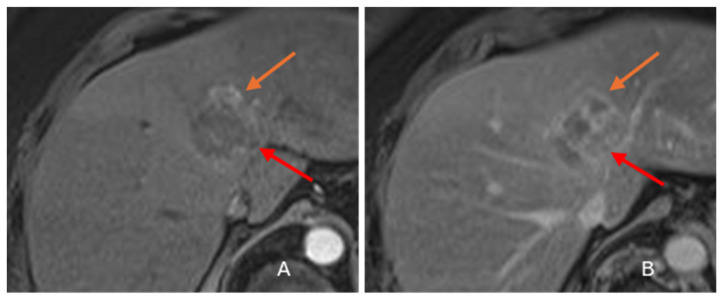
Axial gadolinium-enhanced MRI images in an 82-year-old female with F3 to 4 fibrosis and a pathologically proven cHCC-CC on resection. A 4 cm cHCC-CC in segment 4A shows overlapping features of both HCC and ICC with two distinct foci of differing enhancement within the tumor. A 1.2 cm focus at the 11 to 1 o’clock position (**orange arrows**) shows LR5 features, including heterogeneous arterial enhancement (**A**), followed by delayed phase washout (**B**). A 2.4 cm focus at the 2 to 5 o’clock position (**red arrows**) shows LR M features, including peripheral arterial enhancement (**A**), followed by delayed central progression (**B**).

Due to its biphenotypic composition, cHCC-CC contains varying proportions of both HCC and ICC. Tumors with a greater HCC component (‘HCC predominant cHCC-CC’) may exhibit LR 4/5 features mimicking HCC [Figure 3] [10,22,29,30]. Those with a greater ICC component (‘ICC predominant cHCC-CC’) show LR M features mimicking ICC [Figure 4] [30]. Zhou et al. reported that 45.8% of cHCC-CCs could be categorized as LR 5 and 41.7% as LR M on MRI [31]. Due to these issues, imaging misdiagnosis may occur in at least two-thirds of cHCC-CCs [32]. cHCC-CC with roughly equal proportions of HCC and ICC elements may show overlapping imaging features of both tumors [Figure 5]. This mixed enhancement pattern may include both washout and persistent enhancement in different regions of the tumor, a feature reported to have a 48% sensitivity and 81% specificity for cHCC-CC [26]. Additionally, the main imaging pattern in cHCC-CC, favoring either LR M or LR 4/5, has implications beyond diagnosis. Studies have reported that cHCC-CCs with LR M features have more aggressive biologic behavior and worse outcomes than those with LR 4/5 features [33,34,35]. This varying aggressiveness is probably related to the dominant tissue component (cholangiocytic vs. hepatocytic) [29,36,37,38].

Based on an analysis of 29 cases, Wells et al. proposed that cHCC-CC may exhibit 4 distinct CT/MRI enhancement patterns [39]. Type 1—a HCC enhancement pattern, observed in 6.9%. Type 2—an ICC enhancement pattern, found in 79.3%. Type 3—peripheral hyperenhancement on both the arterial and delayed phases, seen in 6.9%. This appearance is atypical for HCC, and may be seen in ICC, necrotic tumors or infection. Type 4—two separate regions of differing enhancement within the same tumor mass, with one region showing late arterial enhancement and the other showing delayed enhancement. Observed in 6.9%, this appearance is suggestive of either a biphenotypic neoplasm or two distinct adjacent neoplasms (collision tumor).

In an MRI study of 33 cHCC-CCs, Sammon et al. observed that cHCC-CCs showed an HCC enhancement pattern with washout in 39.4%, and an ICC enhancement pattern with progressive enhancement in 39.4% [40]. Additionally, 9% of cHCC-CCs demonstrated an overlapping HCC and ICC enhancement pattern with simultaneous washout and progression [40].

In a CT/MRI study of 54 cHCC-CCs, 41 HCCs, and 41 ICCs, Wang et al. observed that cHCC-CCs showed an HCC enhancement pattern (washin and washout) in 46.3%, an ICC enhancement pattern (progressive enhancement) in 22.2%, a mixed enhancement pattern in 22.2%, and persistent enhancement in 9.3% [41]. The authors suggested that small tumors (<3 cm) may behave like HCC or ICC without significant overlap, with intermediate-sized tumors (3 to 5 cm) showing more HCC-like features, and larger tumors being more likely to show features of both HCC and ICC [41]. In the study, the frequencies of cirrhosis (72.2% vs. 87.8% vs. 7.3%) and intratumoral vessels (63% vs. 85.4% vs. 17.1%) were comparable between cHCC-CCs and HCC, but lower in ICC (*p* < 0.05) [41]. Poorly defined tumor margins (79.6% vs. 82.9% vs. 31.7%) and regional adenopathy (31.5% vs. 48.8% vs. 2.4%) were comparable between cHCC-CCs and ICC, but lower in HCC (*p* < 0.05) [41]. A pseudocapsule was present in 37% of cHCC-CCs, 85.4% of HCCs, and 2.4% of ICCs (*p* < 0.001) [41]. Capsular retraction was found in 24.1% of cHCC-CCs, 56.1% of ICCs and 2.4% of HCCs (*p* ≤ 0.003) [41]. Biliary dilation was observed in 24.1% of cHCC-CCs, 61% of ICCs and 0% of HCCs (*p* < 0.001) [41]. Portal vein thrombosis occurred in 18.5% of cHCC-CCs, 31.7% of HCCs, and 4.9% of ICCs (*p* < 0.001) [41]. The frequency of satellite lesions was comparable: 24.1% in cHCC-CC, 17.1% in HCC, and 22% in ICC (*p* = 0.707) [41].

Gd-EOB-DTPA MRI may provide significant benefits for lesion characterization. For small liver lesions (1 to 3 cm) in cirrhotic patients undergoing imaging surveillance, Granito et al. observed that the presence of double hypointensity in both the portal and hepatobiliary phases, without accompanying arterial enhancement, is a highly suggestive feature of hypovascular HCC [42]. Additionally, Park et al. found that MR findings of cHCC-CCs on Gd-EOB-DTPA correlated with pathological features and prognosis. cHCC-CCs that were hypervascular on the arterial phase had a larger HCC component vs. ICC component, and a better postoperative survival [43].

The diagnosis of cHCC-CC may be improved by integrating imaging findings with serum biomarkers. In a study of 54 cHCC-CC and 55 HCCs, Zhou et al. proposed the following diagnostic criteria for cHCC-CC: (a) LR M on CT/MRI, with arterial phase hyperenhancement on contrast-enhanced ultrasound (CEUS), (b) LR 5 on CT/MRI or CEUS, with an elevated serum CA 19-9, (c) LR M on CT/MRI or CEUS, with an elevated serum AFP [44]. The study found that the criteria above had 64.8% sensitivity, 84.4% specificity, 76.1% accuracy, and an area under the curve (AUC) of 0.75 for detecting cHCC-CC [44].

As a final takeaway, important indicators for the presence of cHCC-CCs include (a) synchronous elevation of serum tumor AFP and CA19-9, (b) a mixed or atypical enhancement pattern on imaging, and (c) discordance between the imaging findings and serum tumor markers (e.g., a HCC imaging pattern with elevated serum CA19-9 or an ICC pattern with elevated serum AFP) [18,39,40,45]. These cases warrant further investigation with biopsy. Imaging alone has a 48% sensitivity and 81% specificity for cHCC-CC [16,26]. Adopting a two-step strategy of sequential imaging and biopsy can increase sensitivity to 60% and specificity to 82% [16,26]. According to Mao et al., the correlation between imaging and histological findings in cHCC-CCs is approximately 66.7% [38].

## 5. Treatment Options

This section discusses treatment options. At present, there are no national or international guidelines that provide a standardized treatment policy for cHCC-CC [46].

### 5.1. Liver Resection

Liver resection is the mainstay of treatment for potentially curative cHCC-CC [46]. The goal is to excise the tumor while maintaining adequate surgical margins and preserving sufficient functional liver volume [14,46]. An anatomic resection along with hilar nodal dissection is recommended as cHCC-CC may exhibit biologic characteristics similar to both HCC and ICC, with HCC showing a predilection for portal vein invasion and ICC having a tendency for hilar lymph node spread [13,18]. Overall survival rates following liver resection for cHCC-CC are outlined in Table 2 [47,48,49,50,51]. This is less favorable than HCC and approaches that of ICC [52,53,54]. Prognostic factors for resected cHCC-CC include tumor size > 5 cm, resection margin < 2 cm, tumor multifocality, higher tumor stage, incomplete capsule formation, satellite nodules, vascular invasion, lymph node involvement, and serum CA 19-9 elevation [18,52,55,56,57,58,59].

### 5.2. Liver Transplantation

Liver transplantation is an established treatment option for HCC within Milan criteria [60,61,62,63]. In contrast, it is seldom performed for ICC due to poor outcomes [1,60,64,65]. The role of transplantation for cHCC-CC, which contains elements of both HCC and ICC, remains unclear. There is controversy in the medical literature and within the transplant community [46,66]. Early studies, many retrospective and small in size, indicate poor outcomes for cHCC-CC, including higher post-transplant recurrence rates and lower survival compared to HCC [52,53,67,68,69,70,71,72]. More recent studies comparing post-transplant survival between cHCC-CC and HCC are outlined in Table 3. Often, the diagnosis of cHCC-CC is only determined at explant, with most tumors being misdiagnosed preoperatively as HCC [66,68,72,73,74]. Institutions are hesitant to extend transplantation to patients with known cHCC-CC due to perceived underwhelming outcomes and donor shortages. With new technologies, the donor pool is expanding, which may provide an opportunity for broadening recipient diagnostic criteria [75,76]. For cHCC-CC patients with cirrhosis, the only other surgical option is liver resection. However, those with large tumors may not be candidates for resection due to limited functional liver reserve [66]. Moreover, recent studies suggest that transplantation should not be dismissed in well-selected patients and call for a nuanced approach [1,66,73].

Vichez et al. analyzed transplant outcomes from information obtained from the United Network for Organ Sharing [60]. Between 1994 and 2013, a total of 4049 patients underwent liver transplantation in the United States, of which 3515 (86.8%) had HCC, 440 (10.9%) had ICC, and 94 (2.3%) had cHCC-CC. The data showed that transplant recipients with cHCC-CC had lower overall survival than those with HCC (*p* = 0.002) [Table 3]. Additionally, the survival rates for cHCC-CC were similar to those for ICC (79% at 1 year, 58% at 3 years, and 47% at 5 years). The median survival following transplantation for cHCC-CC was 29 months.

Gentile et al. performed a systematic review of 14 studies that included 13,613 patients with primary liver cancer [53]. Liver transplantation was performed on 36 patients with cHCC-CC and 404 patients with HCC. At a mean follow-up of 35.6 months, tumor recurrence was found in 36.1% of patients with cHCC-CC compared with 7.7% with HCC. 61.5% of cHCC-CC recurrences were extrahepatic, while 38.5% were intrahepatic. The recurrence-free survival (*p* = 0.001) and overall survival (*p* = 0.018) were lower for cHCC-CC compared to HCC [Table 3].

Kim et al. compared transplant outcomes for cHCC-CC vs. HCC across 9 institutions [74]. 70 patients with cHCC-CC were propensity-matched 1:1 to patients with HCC based on preoperative variables. The findings showed that recurrence-free survival and overall survival were significantly lower in cHCC-CC than in HCC [Table 3]. Extrahepatic recurrence was observed in 75.5% of patients with cHCC-CC, compared with 33.3% in HCC (*p* < 0.001). On multivariate analysis, predisposing factors for tumor recurrence included cHCC-CC (Odds Ratio, OR 2.53, 95% CI 1.19 to 5.38) (*p* = 0.02), microvascular invasion (OR 3.23, 95% CI 1.49 to 7.03) (*p* = 0.003), and number of locoregional therapies before transplantation > 3 (OR 2.33, 95% CI 1.24 to 4.39) (*p* = 0.009). Factors associated with mortality included cHCC-CC (OR 2.28, 95% CI 1.06 to 4.92) (*p* = 0.036), tumor size > 3 cm (OR 1.34, 95% CI 1.12 to 1.61) (*p* = 0.001), and tumor number > 3 (OR 1.03, 95% CI 1.00 to 1.06) (*p* = 0.026).

Lunsford et al. also evaluated transplant outcomes after propensity-matching [66]. 12 patients with cHCC-CC were matched 1:3 to two separate cohorts of 36 patients with HCC. Propensity-matching was performed for (a) pre-transplant tumor characteristics, including radiologic diameter and serum AFP, and (b) explant tumor characteristics, including pathologic diameter, grade/differentiation, and vascular invasion. Matched on pre-transplant characteristics, cHCC-CC showed higher tumor grade and poorer differentiation than HCC but had similar T stage and vascular invasion rates. Patients with cHCC-CC trended toward higher recurrence than those with HCC (50% vs. 22%) and shorter median time to recurrence (297 days vs. 347 days), *p* = 0.07. However, recurrence-free survival and overall survival were not significantly different between cHCC-CC and HCC after propensity-matching [Table 3]. All 6 cHCC-CC recurrences occurred exclusively in poorly differentiated tumors. The authors concluded that low-grade and well-moderately differentiated cHCC-CCs have good post-transplant survival and a low risk of recurrence.

Dageforde et al. conducted an analysis across 12 institutions evaluating surgical outcomes for cHCC-CC and HCC [1]. For cHCC-CC, a comparison between transplantation (*n* = 99) and resection (*n* = 109) showed that transplanted patients had better recurrence-free survival (*p* < 0.001) and overall survival (*p* = 0.047), regardless of tumor burden. On multivariate analysis, tumor burden within Milan criteria (Hazard Ratio, HR = 0.53, 95% CI 0.34 to 0.84) was the only factor associated with improved survival (*p* = 0.007). Additionally, both transplantation (HR = 0.5, 95% CI 0.25 to 1.02) (*p* = 0.047) and tumor burden within Milan criteria (HR = 0.51, 95% CI 0.31 to 0.83) (*p* = 0.007) were identified as factors for lower disease recurrence. In the transplant cohort comprising 99 patients with cHCC-CC and 2174 patients with HCC, cHCC-CC was associated with lower recurrence-free and overall survival, and higher recurrence rates than HCC (*p* < 0.007). However, for transplants performed within the Milan criteria (67 cHCC-CC and 1814 HCCs), there were no significant differences in recurrence-free survival (*p* = 0.74) or overall survival (*p* = 0.81) between cHCC-CC and HCC [Table 3]. Nevertheless, cHCC-CC had a higher recurrence rate compared to HCC (23.1% vs. 11.5% at 5 years; *p* < 0.001).

Finally, a study by Jung et al. suggested that liver transplantation is a viable option in patients with cHCC-CC who met strict selection criteria [73]. Data from 32 transplant recipients with cHCC-CC revealed a 5-year overall survival of 93.3% and a low recurrence rate (13.3%) in patients with early tumors (1 to 2 nodules ≤ 2 cm).

### 5.3. Locoregional Treatments

**A.**  **Transarterial Chemoembolization (TACE)**

TACE has been proposed as a treatment option to extend survival in patients with advanced disease not amenable to surgery [46]. In TACE, a concentrated dose of chemotherapeutic agents is selectively delivered to cancer tissue while sparing the uninvolved liver and reducing systemic side effects [77]. The use of embolic agents also deprives cancer tissue of its blood supply [77]. However, very few studies have evaluated the clinical effectiveness of TACE in patients with cHCC-CC. The largest study by Kim et al. involved 50 patients who underwent TACE for non-resectable cHCC-CC between 1997 to 2009 [37]. In the study, 70% of patients showed a partial response (≥30% reduction in maximum tumor diameter) or stable disease (>50% tumor necrosis despite no significant decrease in tumor size). Conversely, 30% of patients exhibited progressive disease (≥20% increase in the maximum tumor diameter). The median survival was 12.3 months (95% CI, 6.7 to 17.9 months) with survival rates of 52% at 1 year, 38% at 2 years, 16% at 3 years, and 12% at 4 years. On multivariate analysis, prognostic factors for survival following TACE included portal vein invasion (HR 6.45, 95% CI 2.72 to 15.30, *p* < 0.001), Child Pugh B class (HR 4.30, 95% CI 1.89 to 9.77, *p* = 0.001), hypovascular tumors (HR 4.19, 95% CI 1.79 to 9.79, *p* = 0.001), and tumor size ≥ 9 cm (HR 2.49, 95% CI 1.11 to 5.60, *p* = 0.028). A common perception is that cHCC-CC is less vascular and more fibrotic than HCC due to its cholangiocellular component [37,78,79]. However, 40 of 50 patients (80%) demonstrated arterial phase tumor vascularity, resulting in post-TACE tumor response in 34 patients. In contrast, hypovascular cHCC-CC had a lower response rate (10% vs. 85%, *p* < 0.001) and shorter median survival (4 months vs. 16 months) than hypervascular cHCC-CC. The poorer outcomes may be due to diminished delivery of chemoembolic material to hypovascular tumors or because these tumors are inherently more aggressive. A separate study evaluating TACE in recurrent cHCC-CC (*n* = 42) post-resection found that tumors with global arterial enhancement had higher treatment response rates (36% vs. 0%, *p* = 0.005) and longer median survival (52.8 months vs. 12.4 months, *p* < 0.05) compared to tumors with peripheral/rim enhancement or iso-enhancement [80]. The findings suggest that tumor vascularity can be used to identify patients who may benefit from TACE.

**B.**  **Transarterial Radioembolization (TARE)**

TARE utilizing yttrium-90 has shown promise in treating hypovascular liver tumors, including ICC and hepatic metastases [37,81,82]. Its effectiveness for cHCC-CC is under investigation. A study by Malone et al. evaluated the outcomes of TARE in 22 patients with unresectable cHCC-CC between 2012 to 2018 [83]. The study reported complete treatment response in 15%, partial response in 40%, stable disease in 10%, and progressive disease in 35%. Notably, 1 patient was downstaged to resection, while 2 were bridged to transplantation. The median survival was 9.3 months (range 2.5 to 31 months). Factors associated with reduced survival included treatment non-response, multiple tumors, bilobar disease, and serum CA 19-9 elevation. In a study by Bader et al., outcomes following TARE were compared between propensity-matched cohorts with cHCC-CC and HCC [84]. Propensity-matching based on baseline clinical characteristics was performed 1:1, with 10 patients per cohort. The study found no significant differences in disease-free or overall survival between the cHCC-CC and HCC cohorts following TARE. The median disease-free survival was 15.2 months (95% CI 2.7 to 20.2 months) for cHCC-CC vs. 11.6 months (95% CI 2.5 to 19.3 months) for HCC (*p* = 0.94). The median overall survival was 15.2 months (95% CI 2.7 to 20.2 months) for cHCC-CC vs. 12.3 months (95% CI 6 to 17.4 months) for HCC (*p* = 0.98). Lastly, a study by Fowler et al. compared outcomes in patients with cHCC-CC who underwent TARE (*n* = 6) vs. TACE (*n* = 6) [85]. For TARE vs. TACE, 50% vs. 20% showed partial response, 0% vs. 40% had stable disease, and 50% vs. 40% showed progressive disease. The mean disease-free survival was 8.3 months, while the overall survival was 16 months.

**C.**  **Ablation**

Local ablative therapies, such as radiofrequency ablation and microwave ablation, are established treatment options for small HCCs (≤3 cm). However, there is no granular information regarding the effectiveness of ablative therapies for cHCC-CC. Analysis of data from the National Cancer Data Base from 2004 to 2015 found that a total of 77 patients with cHCC-CC underwent ablation [86]. The 5-year overall survival following ablation for cHCC-CC was 18.9% (95% CI 9.8 to 30.3%). In comparison, the 5-year overall survival following ablation for HCC (*n* = 14,064) and ICC (*n* = 499) were 30.5% (95% CI 29.5 to 31.5%) and 17.3% (95% CI 13.1 to 22%) respectively, (*p* = 0.01%).

### 5.4. Systemic Therapy

Patients with unresectable disease, including those with metastases, may be candidates for systemic therapy. However, there are no standard treatment regimens for cHCC-CC, and evidence supporting the effectiveness of systemic treatment is limited. In general, drugs used for HCC, such as multi-kinase inhibitors, or ICC, such as platinum-based therapy and immune checkpoint inhibitors, have been adopted for cHCC-CC. Table 4 presents outcomes following systemic therapy for cHCC-CC [87,88,89,90,91,92]. The findings demonstrate consistently poor recurrence-free and overall survival.

## 6. Current/Future Directions: Radiomics AI

Over the last decade, cutting-edge advances in computer technology have driven exponential growth and activity in radiomics AI. Sophisticated machine-learning algorithms have been developed and iteratively refined to extract high-throughput quantitative imaging features from medical images that are invisible to the human eye. This facilitates a comprehensive, objective analysis of disease processes by quantifying radiomic features, such as tissue texture and shape, using modern data analytics. Radiomics has been evaluated in primary liver cancers to address challenges in improving tumor diagnosis, assessing treatment response, and predicting patient outcomes. Additionally, AI can uncover hidden correlations between radiomic features and biologic phenotypes, enhancing our understanding of disease processes and offering fresh insights. The use of radiomics for (a) differentiating cHCC-CC from other primary liver cancers, and (b) predicting post-treatment recurrence and survival is the focus of this section. While early publications show promising results, significant limitations remain. Most reported studies were conducted retrospectively and were prone to selection bias. Most studies were conducted at a single center and lacked external validation. The sample sizes were small, as large numbers were difficult to obtain due to the rarity of cHCC-CC. Methodologies, scanning protocols, and imaging platforms varied across studies, resulting in heterogeneous datasets and challenging the generalizability of findings. Many studies analyzed only patients who underwent surgical resection for curative intent, skewing data toward patients with less aggressive disease and lower tumor burden. Incorporating radiomics into the clinical workflow remains a challenge due to high technical and infrastructure requirements.

**A.**  **Differentiation of cHCC-CC from other primary liver cancers**

Deng et al. developed a machine-learning model using clinical and MRI data to differentiate cHCC-CC from HCC preoperatively [93]. The study involved 52 patients with cHCC-CC and 142 with HCC. The authors found that a combined clinical-radiomics model performed best, with an AUC of 0.88 (95% CI, 0.77 to 0.95), sensitivity of 0.71, and specificity of 0.84. In comparison, the radiomics model had an AUC of 0.84 (95% CI 0.72 to 0.92), sensitivity of 0.57, and specificity of 0.87. The lowest performance was observed for the clinical model, with an AUC of 0.76 (95% CI 0.66 to 0.86), sensitivity of 0.57, and specificity of 0.73. Compared with HCC, multiple tumors, irregular edges, and peritumoral arterial hyperenhancement were more common in cHCC-CC (*p* < 0.05). Conversely, the prevalence of cirrhosis and intralesional lipid was higher in HCC (*p* < 0.05).

Guo et al. conducted a radiomics analysis of preoperative clinical and MRI data to differentiate cHCC-CC from HCC [30]. The study involved 125 cHCC-CC patients and 217 HCC patients who underwent a hepatectomy. The study found that a combined clinical-radiomics model performed best, with an AUC of 0.90 (95% CI 0.86 to 0.93), a sensitivity of 0.74, and a specificity of 0.92. The radiomics model performed second-best, with an AUC of 0.86 (95% CI 0.81 to 0.90), a sensitivity of 0.69, and a specificity of 0.93. Finally, the clinical model performed worst, with an AUC of 0.77 (95% CI 0.71 to 0.82), sensitivity of 0.60, and specificity of 0.85.

To differentiate cHCC-CC from ICC, Zhou et al. developed a combined clinical-radiomics model that incorporated clinical variables like serum AFP and background liver disease, such as cirrhosis or chronic hepatitis, along with an MRI radiomics model [94]. The study involved training (45 cHCC-CC and 106 ICC) and validation (19 cHCC-CC and 46 ICC) cohorts. The authors found that the combined model achieved an AUC of 0.95 (95% CI 0.9 to 0.98) in the training group and 0.90 (95% CI 0.80 to 0.96) in the validation group. The combined clinical-radiomics model demonstrated superior diagnostic performance compared with either the radiomics or clinical models (*p* < 0.05). Concordant with others, the authors observed that patients with cHCC-CC were more likely to have higher serum AFP levels, background liver disease, were more often males and <60 years of age, compared to those with ICC [11,20,41,95].

To differentiate cHCC-CC from HCC and ICC, Liu et al. developed a machine-learning model using MRI and CT radiomics features in a patient cohort comprising 24 cHCC-CCs, 38 HCCs, and 24 ICCs [96]. The study found that MRI had the highest performance for differentiating cHCC-CC from HCC and ICC, with an AUC of 0.77 ± 0.19. In comparison, CT performance was modest, with an AUC of 0.64 ± 0.17. For differentiating HCC from cHCC-CC and ICC, radiomic features derived from contrast-enhanced MRI, non-contrast CT, or portal-phase CT showed good diagnostic performance, with AUCs ranging from 0.79 ± 0.07 to 0.81 ± 0.13 for MRI, 0.81 ± 0.06 for non-contrast CT, and 0.71 ± 0.15 for portal-phase CT. The study found that radiologists’ ability to differentiate cHCC-CC on qualitative imaging assessment was poor. cHCC-CC was misdiagnosed as HCC or ICC in 58% of MRIs and 69% of CTs. The variable imaging findings in cHCC-CC, which resemble HCC or ICC depending on the proportion of hepatocellular or cholangiocellular components, and radiologists’ limited experience with this rare tumor, are the main contributors to poor performance.

Finally, Chen et al. developed an ultrasound-based deep learning model in 465 patients, including 96 cHCC-CCs, 264 HCCs, and 105 ICCs [97]. Four deep convolutional neural network models—ResNet18, InceptionV3, MobileNet, and DenseNet121—were assessed. The study found that ResNet18 achieved the best results, with an AUC of 0.92 (95% CI 0.86 to 0.98), sensitivity of 0.85, and specificity of 0.93 for differentiating the three tumor types. In the study, the authors elected to use only the most representative B-mode US image of the tumor in the deep learning model. They did not use data from cine clips, and the images were uploaded in JPEG format rather than DICOM. Future studies should look at optimizing these parameters.

**B.**  **Prediction of post-treatment recurrence and survival**

Authors have investigated whether radiomics can predict prognosis following surgery, including the risk of recurrence and overall survival. This information is important for prognostication, differentiating patients into high- and low-risk categories, and identifying those that benefit most from surgery.

Tang et al. developed an integrative normogram to predict overall survival using data from 118 cHCC-CC patients who underwent hepatectomy [98]. CT radiomics features were integrated with clinical data to create the normogram. The radiomics features included NGLDM Busyness, MeanValue, and GLZLM HGZE. Clinical features included the bilirubin level, vascular invasion, anatomic resection, and satellite lesion(s). For predicting overall survival, the integrative normogram performed the best (HR of 8.16, 95% CI 4.50 to 14.79), followed by the radiomics score (HR 5.91, 95% CI 3.29 to 10.63), and then the clinical model (HR 2.65, 95% CI 1.53 to 4.60), *p* < 0.001. The integrative normogram was associated with an AUC of 0.88 and 0.89 for 1-year and 3-year survival, respectively. Patients identified as high-risk had lower overall survival than those at low risk (median of 6.1 months vs. 81.6 months, *p* < 0.001). Overall survival at 1 year was 10.8% for the high-risk group vs. 84% for the low-risk group (*p* < 0.001). At 3 years, the overall survival was 2.7% for the high-risk group vs. 69.1% for the low-risk group (*p* < 0.001).

There is increasing evidence that microvascular invasion (MVI) is a key prognostic factor for disease recurrence [99,100,101,102]. Several investigators have evaluated radiomics models for predicting MVI. Zhou et al. constructed a predictive model based on 4 MR radiomic features in a cohort of 91 cHCC-CC patients [100]. The authors found that larger tumor size and a higher Radscore score were associated with MVI in both the training (*p* = 0.026 and *p* < 0.001, respectively) and validation (*p* = 0.04 and 0.001, respectively) cohorts. The radiomic model showed good diagnostic performance for predicting MVI, with AUCs of 0.87 (95% CI 0.76 to 0.94, *p* < 0.001) in the training cohort and 0.84 (95% CI 0.65 to 0.95, *p* < 0.001) in the validation cohort.

Xiao et al. evaluated combined clinical and imaging findings, and MRI radiomics in a study of 143 cHCC-CC patients [103]. On logistic regression, the only clinical and imaging features that were significant MVI predictors were tumor size (OR 2.04, 95% CI 1.21 to 3.78) (*p* = 0.015) and surface retraction (OR 4.69, 95% CI 1.25 to 22.84) (*p* = 0.03). The radiomics model showed good predictive performance for MVI, comparable to the combined clinical-imaging-radiomic model, in the training (*p* = 0.87), validation (*p* > 0.99), and test (*p* = 0.85) cohorts. The AUC for the radiomics model was 0.94 (95% CI 0.89 to 0.99) in the training set, 0.87 (95% CI 0.76 to 0.99) in the validation set, and 0.78 (95% CI 0.58 to 0.98) in the test set. The combined clinical-radiomic model had an AUC of 0.94 (95% CI 0.89 to 0.99) in the training set, 0.87 (95% CI 0.75 to 0.99) in the validation set, and 0.79 (95% CI 0.59 to 0.98) in the test set. Overall survival was lower in the predicted MVI-positive cohort (median 18 months) compared to the predicted MVI-negative cohort (median 25 months) (*p* = 0.008).

## 7. Conclusions

cHCC-CC is a biphenotypic primary liver cancer containing a variable proportion of HCC and ICC elements. Depending on its dominant tissue component, it may exhibit HCC- or ICC-like features, while tumors containing roughly equal proportions of both components may show overlapping or mixed features. cHCC-CC remains a challenge to diagnose and treat. Due to its rarity, clinicians have limited experience with this tumor, and there are no standardized diagnostic algorithms or treatment strategies specific to cHCC-CC. Radiomics AI can aid clinicians in addressing challenges in lesion differentiation from HCC and ICC, and in predicting post-treatment survival and recurrence. Future efforts should also focus on establishing international collaborations to create global registries that facilitate the accumulation and evaluation of prospective data from larger patient cohorts. Additionally, the development of specialized international centers of expertise in cHCC-CC is vital for advancing knowledge and progress in both diagnosis and treatment.

## Figures and Tables

**Table 1:** LI-RADS 2018 Criteria for LR 4/5 and LR M on CT/MRI [28].

**LR 4** Major features • Enhancing capsule • Non-peripheral washout • Threshold growth	**For liver observations with no arterial phase hyperenhancement** • Size < 20 mm plus ≥ 2 major features • Size ≥ 20 mm plus ≥ 1 major feature **For liver observations with non-rim arterial enhancement** • Size < 10 mm plus ≥ 1 major feature. • Size 10–19 mm plus an enhancing capsule. • Size ≥ 20 mm.
**LR 5** Major features • Enhancing capsule • Non-peripheral washout • Threshold growth	**For liver observations with non-rim arterial hyperenhancement** • Size 10–19 mm plus ≥ 1 of non-peripheral washout or threshold growth. • Size ≥ 20 mm plus ≥ 1 major feature.
**LR M**	**For liver observations with at least 1 targetoid or non-targetoid feature, not meeting criteria for LR-5 or tumor in vein** **Targetoid features** • Rim arterial phase hyperenhancement. • Peripheral washout. • Delayed central enhancement. • Targetoid diffusion restriction. • Targetoid transitional phase or hepatobiliary phase intensity. **Non-targetoid features** • Infiltrative appearance. • Necrosis or severe ischemia. • Marked diffusion restriction. • Other imaging features that, in the radiologist’s judgment, suggest a non-HCC malignancy, such as liver surface retraction or adjacent biliary dilatation.

**Table 2:** Overall Survival (OS) following Liver Resection for cHCC-CC.

Study	Country	Patients	OS at 1 Year	OS at 3 Years	OS at 5 Years
Peng et al. [47] 2023	USA	183	76.1%	44.5%	42.7%
Zhang et al. [48] 2022	China	95	73.9%	38.2%	23.6%
Kim et al. [49] 2021	South Korea	153	92.1%	70.9%	61.7%
Zhou et al. [50] 2021	China	206	78%	56%	44%
Wakizaka et al. [51] 2019	Japan	28	57.9%	33.4%	25.1%

**Table 3:** Post-Transplant Survival for cHCC-CC vs. HCC.

Study	Country	Survival for cHCC-CC vs. HCC
Vichez et al. [60] 2016	USA	- OS of 82% vs. 86% at 1 year, 47% vs. 72% at 3 years, 40% vs. 62% at 5 years (*p* = 0.002)
Gentile et al. [53] 2020	Multinational	- RFS of 69% vs. 93.6% at 1 year, 58.4% vs. 92.2% at 3 years, 40.9% vs. 87.4% at 5 years (*p* ≤ 0.037) - OS of 84% vs. 95.4% at 1 year (*p* = 0.055), 65.9% vs. 83.7% at 3 years (*p* = 0.05), 49.4% vs. 80.3% at 5 years (*p* = 0.018)
Kim et al. [74] 2023	South Korea	- RFS of 77.6% vs. 88.3% at 1 year, 62% vs. 82.4% at 2 years, 56.3% vs. 80.6% at 3 years (*p* = 0.012) - OS of 84.4% vs. 93.6% at 1 year, 75.7% vs. 87.9% at 2 years, 63.8% vs. 84% at 3 years (*p* = 0.015)
Lunsford et al. [66] 2018	USA	Matched on pre-transplant characteristics: - RFS of 66% vs. 76% at 1 year, 42% vs.67% at 3 years, 42% vs. 61% at 5 years (*p* = 0.17) - OS of 75% vs. 88% at 1 year, 54% vs. 68% at 3 years, 42% vs. 65% at 5 years (*p* = 0.13) Matched on explant tumor characteristics: - RFS of 66% vs. 69% at 1 year, 42% vs. 56% at 3 years, 42% vs. 44% at 5 years (*p* = 0.44) - OS of 75% vs. 72% at 1 year, 54% vs. 60% at 3 years, 42% vs. 48% at 5 years (*p* = 0.54)
Dageforde et al. [1] 2021	USA	Transplants performed within the Milan criteria - RFS of 70.1% vs. 70.3% at 5 years (*p* = 0.74) - OS of 70.1% vs. 73.4% at 5 years (*p* = 0.81)

RFS—recurrence-free survival, OS—overall survival.

**Table 4:** Outcomes following systemic therapy for cHCC-CC.

Study	Treatment (*n*)	Total Patients	RFS (Median)	OS (Median)
Tanabe et al. [87] Japan 2025	Lenvatinib (14) Atezolizumab + bevacizumab (7)	21	6.5 months	14.9 months
Kim et al. [88] South Korea 2021	Sorafenib (62) Platinum-containing therapy (28) Gemcitabine (1) Fluoropyrimidines (8)	99	3.8 months	10.6 months
Kobayashi et al. [89] Japan 2018	Gemcitabine + cisplatin (12) 5-fluorouracil (11) Sorafenib (5)	28	2.8 months	8.9 months
Salimon et al. [90] France 2018	Gemcitabine + oxaliplatin (18) Gemcitabine + oxaliplatin + bevacizumab (9) Gemcitabine + cisplatin (3)	30	9 months	16.2 months
Rogers et al. [91] USA 2017	Gemcitabine + cisplatin (1) Gemcitabine + bevacizumab (2) Gemcitabine (1) Sorafenib (3)	7	3.4 months	8.3 months
Connell et al. [92] USA 2015	Gemcitabine or 5-fluorouracil (5) Platinum-containing therapy (6) Platinum-containing therapy + sorafenib (6) Sorafenib (8)	25	2.3 months	10 months

RFS—recurrence-free survival, OS—overall survival.

## Data Availability

No new data were created or analyzed in this study. Data sharing is not applicable to this article.
